# Degree and Predictors of Functional Loss of the Operated Kidney following Nephron-Sparing Surgery: Assessment by Quantitative SPECT of 99m Tc-Dimercaptosuccinic Acid Scintigraphy

**DOI:** 10.1155/2011/961525

**Published:** 2011-08-10

**Authors:** Ofer Nativ, Amos Levi, Roy Farfara, Sarel Halachmi, Boaz Moskovitz

**Affiliations:** Department of Urology, Bnai Zion Medical Center, 47 Golomb St., Haifa 31048, Israel

## Abstract

*Purpose*. To determine the degree and predictors of renal function loss of the operated kidney following nephron-sparing surgery (NSS). *Material and methods*. The study group included 113 patients with renal mass who underwent NSS at our institution. QDMSA before and 3–6 months after surgery was used for evaluation differences in renal function of each kidney. Mean change of percent uptake by the kidney was correlated with various clinical and pathological variables. *Results*. The overall average decrease of renal function of the operated kidney as measured by QDMSA was 10.5% ± 2.6 SER. Among the studied variables, the most important predictors of postoperative ipsilateral residual kidney function were estimated blood loss (EBL), *P* = 0.0003, duration of warm ischemia, *P* = 0.008, patient's age at surgery, *P* = 0.024, method used for tumor bed closure, *P* = 0.06, and location of the lesion, *P* = 0.08. *Conclusions*. Carful hemostasis, minimal duration of arterial clamping, and use of tissue adhesives to seal tumor bed are associated with maximal preservation of postoperative residual renal function after NSS. These variables should be considered by the operative team when planning the surgical procedure .

## 1. Introduction

Nephron-sparing surgery (NSS) is now considered as the preferable treatment for most patients with organ-confined renal cancer. Such approach provides excellent cancer control comparable to that obtained after radical nephrectomy with the advantage of renal function preservation [1, 2]. There are increasing data in the literature that demonstrate association between postoperative renal function and noncancer mortality mainly due to cardiovascular events [3, 4]. For this reason, prevention of functional tissue damage during NSS is crucial for patients' survival.

Most studies evaluate postoperative renal function using serum creatinine level or by estimated glomerular filtration rate (eGFR). For patients with two kidneys, these methods underestimate the true kidney function and cannot consider the contribution of each renal unite (especially the operated one) to the total renal function [5, 6]. In order to assess the contribution of each renal unit to the total renal function, we used the quantitative SPECT of 99m Tc-Dimercaptosuccinic Acid (QDMSA).

Scintigraphy [7] before and after NSS before compensation could occur.

The aim of the current study was to assess the degree and predictors of functional loss of the involved kidney after NSS. 

## 2. Material and Methods

### 2.1. Patients

The study was carried out after achieving the IRB approval. Among all nephron-sparing surgical procedures performed at our institution, we identified 113 patients who had both preoperative and 3–6 months postoperative QDMSA renal scans. There were 66 males and 47 females with mean age of 58.7 years (median: 58 years, range: 28–85 years). Eleven patients had a single kidney and the average serum creatinine before the surgical procedure was 1.11 mg% (range: 0.6–3.4 mg%). For each patient, the following variables were obtained from the medical records: gender, age, operated side, number of kidneys, location of the lesion (peripheral/central), preoperative maximal tumor diameter (obtained from the CT scan), number of lesions, initial serum creatinine, comorbidities (cardiovascular, hypertension, diabetes, urolithiasis, polycystic disease, and chronic renal failure), intraoperative estimated blood loss (EBL), need for blood transfusion, opening of the collecting system, duration of warm ischemia, and type of tumor bed closure (sutures/sealant).

### 2.2. Surgical Technique:

A standard 11th rib flank approach with the patient in full flank position was used. After complete mobilization of the kidney within Gerota's fascia, the ureter and vascular pedicle were identified and isolated on a vascular loop. 0.5 gr′/kg′ mannitol was given intravenously before clamping the renal artery and vein with subsequent cooling of the kidney surface with ice-slush saline for 20 minutes. Enucleation of the tumor involved circumferential incision followed by blunt dissection between the fibrous pseudocapsule and the renal parenchyma. Attention was made to remove a rim of minimal normal renal tissue. Samples from the remaining renal parenchyma at the tumor base were sent for intraoperative frozen section analysis to verify a tumor-free margin. Open blood vessels or collecting system were sutured using monocryl 4/0 continuous sutures; argon beam coagulator was used to seal the exposed renal parenchyma. Tumor bed was closed either with large sutures (1/0 vicryl with blunt-end liver needle) to approximate the edges of the parenchymal defect, or by using 2–10 mL of tissue adhesive (BioGlue Cryolife, Atlanta, Ga) to fill the tumor bed. Pedicle clamping was than removed, warm ischemia time was determined, and the kidney was inspected for bleeding or urinary leakage.

### 2.3. QDMSA Scan

Quantitative SPECT of 99m Tc-DMSA uptake by the kidney was done as described previously [4, 5], the patient was injected with 75–150 MBq (2–4 mCi) 99m Tc-DMSA, and SPECT was performed after 4–6 hours. We used a rotating single head gamma camera with a low-energy collimator (Apex 415-ECT: Elscint, LTD., Haifa, Israel). Data was accumulated from 120 projections 3° apart; the process lasted for about 20 minutes. 

Raw data were reconstructed by filtered back projections with a Hann filter (cutoff point of 0.5 cycle/cm). Following reconstruction, each image was sectioned at 1 pixel (0.68 cm) intervals in transaxial, coronal, and sagital planes using a 64 × 64 byte matrix. 

Kidney volumes and radioactive concentration measurements were calculated on the reconstructed data using the threshold method with a 43% threshold value that is known to best fit the target to nontarget ratio of DMSA in the kidney [6].

Data analysis was automated and operator independent, and the operator chose the slice that best defines the kidney and drew a region of interest (ROI) around it. Volume measurement was calculated by the sum of pixels in all sections multiplied by slice thickness. Concentration measurement used all pixels within the ROI that had a higher reading than the threshold. Counts per voxel were converted into concentration units (MBq/cm^3^[*μ*Ci/cm^3^]) using the regression line obtained by previous phantom measurements. The injected dose density (%ID per cm^3^ of renal tissue) was calculated by using this value corrected for radioactive decay. Multiplying kidney volume with %ID per cubic centimeter gave kidney uptake. Data are presented as change of postoperative kidney function from baseline.

### 2.4. Statistical Analysis

The percent change of QDMSA in the operated kidney (the dependent variable) was calculated as the percent change of the value obtained after surgery considering the preoperative absolute uptake as 100%. 

Samples were analyzed using mean, standard deviation, and standard error. The differences values between subgroups of patients were compared and analyzed by “One-way analysis of variance” (three subgroups or more) or by “*t*-test for differences in means” (pairs of subgroups) to obtain significant differences. *P* value of less than 0.05 was considered statistically significant.

## 3. Results

The overall average decrease of renal function of the operated kidney as measured by QDMSA was 10.5%  ±  2.6 SER.

Among the studied variables, the most influential on postoperative ipsilateral remaining kidney function were estimated blood loss (EBL), duration of warm ischemia, patient's age at surgery, method used for tumor bed closure, and location of the removed lesion (Table 1). All other studied parameters were less important for predicting functional preservation after the surgical procedure.

**Figure 1:**
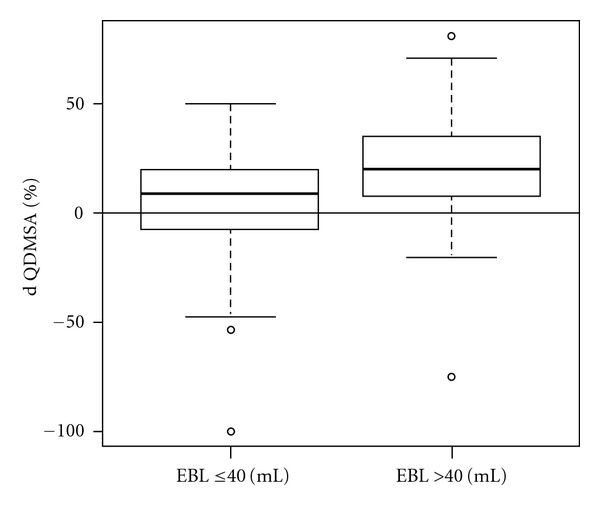
Mean change of postoperative renal function of the operated kidney as measured by QDMSA stratified according to intraoperative estimated blood loss (*P* = 0.0003).

The median blood loss for the studied group was 40 mL, average 153 mL, range 0–3.500 mL. As shown in Figure 1, the mean decrease in postoperative QDMSA uptake of the involved kidney was only 3% for patients with EBL ≤ 40 mL (*n* = 56) compared with 20% for those who had EBL of more than 40 mL, *P* = 0.0003.

**Figure 2:**
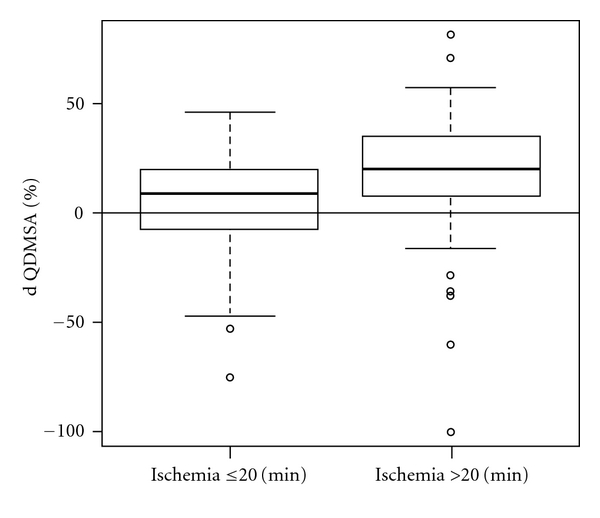
Mean change of postoperative renal function of the operated kidney as measured by QDMSA stratified according to duration of warm ischemia (*P* = 0.008).

Significant association was noted between duration of warm ischemia time and the residual renal function. For kidneys subjected to warm ischemia of more than 20 minutes (*n* = 47), the absolute functional loss was more than fivefold higher compared to kidneys with warm ischemia of 20 minutes or less (QDMSA decrease of 16% versus 3%, resp., *P* = 0.008), Figure 2.

**Figure 3:**
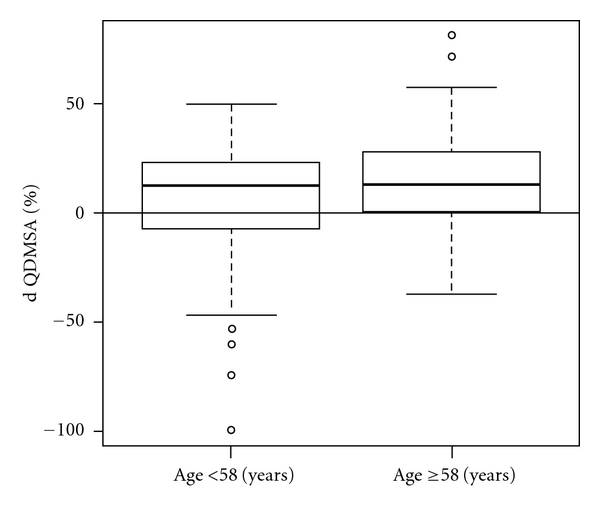
Mean change of postoperative renal function of the operated kidney as measured by QDMSA stratified according to duration of warm ischemia (*P* = 0.024).

Patients' age was found to be a strong predictor of kidney function preservation after tumor removal. The average difference between pre- and postsurgical function for young (58 years or less) and older patients was 5.8% and 16.3%, respectively, *P* = 0.024, (Figure 3).

Analysis of the relationship between percent change in QDMSA change and the method used for closure of tumor bed, that is, traditional sutures (*n* = 74) or BioGlue, revealed reduced functional loss in the later group (4.9% versus 13.4%, resp., *P* = 0.06). Further subgroup analysis for young patients only demonstrated statistically significant differences with 2.3% and 11.2%, resp., decreased ipsilateral renal function, *P* = 0.045).

Peripheral lesions (*n* = 70) were less likely to cause deterioration of the operated kidney QDMSA results comparing centrally located tumors with postsurgery uptake reduction of 7.5% and 15.6%, respectively, *P* = 0.087. 

## 4. Discussion

Recent meta-analysis of 21 studies including more than one million participants has shown that reduced kidney function defined as an estimated glomerular filtration rate (eGFR) less than 60 mL/min/1.73 m² is an independent predictor of all-cause and cardiovascular mortality [7].

Such association is more pronounced in patients with renal cortical tumors for which even in the presence of preoperative normal serum creatinine level, 26% will have eGFR of less than 60 mL/min/m², indicating chronic renal disease [6]. Several studies assessed the relationship between the remaining postoperative renal function and patients' overall survival indicating prognostic advantage for those with higher versus lower eGFR [8, 9]. Weight et al. evaluating overall survival in 1004 patients after surgery for renal cortical tumors using Cox multivariate proportional hazard model of various predicting variables demonstrated that only postoperative eGFR and pathologic stage were statistically significant predictors.

The more frequent use of noninvasive imaging modalities resulted in increased detection of small asymptomatic early-stage renal masses known to be biologically less aggressive [10]. Indeed, many of these patients are more likely to die from noncancer causes than from RCC, emphasizing the need for maximal tissue preservation in patients undergoing NSS [11]. 

In this analysis, we assessed the individual renal function of the operated kidney by means of QDMSA. It is a noninvasive reproducible method for monitoring serial changes in individual renal function [12]. The renal uptake of 99m Tc-DMSA correlates well with the effective renal plasma flow, glomerular filtration rate, and creatinine clearance. Thus, the renal uptake of 99m Tc-DMSA provides a practical index for evaluating individual renal function [13, 14].

In most of the published studies, assessment of postoperative renal function is based on serum creatinine level, 24-hour urinary creatinine clearance, or estimated glomerular filtration rate (eGFR). Such methods can be inaccurate as they depend on gender and body mass and are affected by the status of the contra lateral kidney. The best and most precise approach for evaluation of postoperative renal function of the involved kidney is a radioisotope method such as the QDMSA that we used in our study [15].

The objective of the current study was to identify important variables that can predict postoperative renal function loss with special interest in those that can be controlled in order to improve outcome of future surgical procedures.

Among the tested variables, the most important predictors of decreased renal uptake of the operated kidney were blood loss over 40 mL, warm ischemia time longer than 20 minutes, patients' age of 58 years old or older, suture closure of tumor bed, and central location of the tumor.

It is reasonable to observe association between blood loss and decreased postoperative function. The reduced visibility due to bleeding may result in removal of larger amount of normal tissue. The blood loss may be also related to deeper location of the lesions that are supplied by large-size renal vessels.

Similar findings were also reported by Novick [16] who noted significant decrease (*P* = 0.02) in eGFR among patients who bled more than the average during the procedure. In their experience, for each mL of blood loss, the eGFR decreased by 0.1%.

It has long been recognized that extended warm ischemic time due to renal artery clamping is associated with decreased renal function in a duration-effect direct relationship [17]. We found that 20 minutes of warm ischemia was associated with an average decrease of only 3% of the original kidney function. It is therefore recommended to limit renal pedicle clamping to 20 minutes whenever feasible.

Longer duration of ischemia may significantly compromise the future function of the kidney leading to higher rate of acute renal failure and development of advanced stages of chronic kidney disease. The safety and effectiveness of our cut point time of 20 minutes is in agreement with a recently published international collaborative review of the literature [18]. The practical implication of our finding is that patients who are at increased risk to develop renal failure should be referred to centers with large volume of experienced surgeons who can minimize the duration of clamping of the renal vessels.

Another variable which influenced our ability to preserve renal function was the patients' age at time of surgery. Older patients (≥58 years) had nearly threefold decreased function of the operated kidney compared with that measured for the young group (average postoperative uptake of 83.7% and 94.2%, resp., compared to the initial values, *P* = 0.024). Such finding is expected as we know that age is a strong predictor of chronic kidney disease which is not always detected by existing clinical tests other than renal biopsy. A recent study from the Mayo Clinic that evaluated age impact on rate of nephrosclerosis (glomerulosclerosis, tubular atrophy, interstitial fibrosis, and arteriolosclerosis) among living donors revealed direct association. Among 1203 adults with normal renal function, the frequency of nephrosclerosis was 59.2% for patients older than 60 years but only 24.8% for those 59 year or younger, *P* = 0.001, [19, 20]. It is reasonable to assume that the ischemic damage was more pronounced in kidneys with preoperative damage that was more frequent in older patients who may also be subjected more commonly to comorbidities that can affect kidney function (diabetes, hypertension, cardiovascular disease, or dislipidemia). 

We were able to show that the method used for tumor bed closure is also important (but not statistically significant) for predicting the amount of kidney function loss after tumor removal. The use of BioGlue but not standard sutures was associated with 4.9% versus 13.4% decreased uptake. Separate analysis of young patients demonstrated that the use of tissue adhesives can provide significant preservation of the renal function. The standard technique to close the renal defect following tumor resection is by approximating the transected margins with various suture techniques.

The tension of the sutures on the frail renal parenchyma and the distortion of the normal renal anatomy may result in blood vessels kinking and further ischemic injury of viable tissue. By contrast, the use of tissue sealant, which fills up the cavity in the enucleation site, will result in maximal parenchymal/function preservation [21].

Like the findings of Chan et al. [21], we observed borderline correlation between tumor location and postoperative QDMSA results. Patients with central lesions had twofold decreased function of the operated kidney. This can be explained by the need to excise more normal renal tissue to adequately resect central tumors than peripheral tumors. The likelihood of collecting system repair is higher in deep lesions which also increases the length of warm ischemia time. Moreover, ligation of blood vessels that supply centrally located tumors that are usually larger can disrupt blood supply of uninvolved tissue, resulting in greater permanent ischemic damage of normal renal parenchyma. 

Our study is limited by its retrospective nature, the small study group (113 patients), data obtained from single-institution database, and the absence of other potentially important variables such as body mass index, previous kidney operation, or degree of nephrosclerosis. An advantage of our analysis is the use of QDMSA scan that enabled assessment of the functional loss of the operated kidney without the compensation of the contralateral kidney, and all procedures were performed by a single surgeon. In our model, each patient served as his/her own control which can correct for the small sample size.

## 5. Conclusions

We were able to identify six predictors that are associated with decreased renal function after NSS. Three of these variables are operator dependent (blood loss, warm ischemia, and method used for tumor bed closure) and should be taken into account when performing such surgical procedure. Attempt should be made to reduce intraoperative bleeding, minimize duration of arterial clamping, and use tissue adhesives whenever possible. It is reasonable to consider referral of older patients with centrally located lesions to highly experienced centers.

## Figures and Tables

**Table 1:** Variables affecting the postoperative renal function of the operated kidney after NSS.

Variable	*P*-value
Estimated blood loss (less or grater than 4 mL)	0.0003
Warm ischemia time (more or less than 20 minutes	0.008
Patients' age (younger or older than 58 years)	0.024
Method used for tumor bed closure (suture or BioGlue)	0.06
Location of the lesion (central versus peripheral)	0.087
